# Accumulation of dually targeted StGPT1 in chloroplasts mediated by StRFP1, an E3 ubiquitin ligase, enhances plant immunity

**DOI:** 10.1093/hr/uhae241

**Published:** 2024-08-30

**Authors:** Xintong Wu, Xiaoshuang Zhou, Tianyu Lin, Zhe Zhang, Xinya Wu, Yonglin Zhang, Yanli Liu, Zhendong Tian

**Affiliations:** National Key Laboratory for Germplasm Innovation & Utilization of Horticultural Crops, Huazhong Agricultural University (HZAU), Wuhan 430070, China; Hubei Hongshan Laboratory (HZAU), Wuhan 430070, China; Key Laboratory of Potato Biology and Biotechnology (HZAU), Ministry of Agriculture and Rural Affairs, Wuhan 430070, China; Potato Engineering and Technology Research Center of Hubei Province (HZAU), Wuhan 430070, China; National Key Laboratory for Germplasm Innovation & Utilization of Horticultural Crops, Huazhong Agricultural University (HZAU), Wuhan 430070, China; Key Laboratory of Potato Biology and Biotechnology (HZAU), Ministry of Agriculture and Rural Affairs, Wuhan 430070, China; Potato Engineering and Technology Research Center of Hubei Province (HZAU), Wuhan 430070, China; National Key Laboratory for Germplasm Innovation & Utilization of Horticultural Crops, Huazhong Agricultural University (HZAU), Wuhan 430070, China; Key Laboratory of Potato Biology and Biotechnology (HZAU), Ministry of Agriculture and Rural Affairs, Wuhan 430070, China; Potato Engineering and Technology Research Center of Hubei Province (HZAU), Wuhan 430070, China; National Key Laboratory for Germplasm Innovation & Utilization of Horticultural Crops, Huazhong Agricultural University (HZAU), Wuhan 430070, China; Key Laboratory of Potato Biology and Biotechnology (HZAU), Ministry of Agriculture and Rural Affairs, Wuhan 430070, China; Potato Engineering and Technology Research Center of Hubei Province (HZAU), Wuhan 430070, China; National Key Laboratory for Germplasm Innovation & Utilization of Horticultural Crops, Huazhong Agricultural University (HZAU), Wuhan 430070, China; Key Laboratory of Potato Biology and Biotechnology (HZAU), Ministry of Agriculture and Rural Affairs, Wuhan 430070, China; Potato Engineering and Technology Research Center of Hubei Province (HZAU), Wuhan 430070, China; National Key Laboratory for Germplasm Innovation & Utilization of Horticultural Crops, Huazhong Agricultural University (HZAU), Wuhan 430070, China; Key Laboratory of Potato Biology and Biotechnology (HZAU), Ministry of Agriculture and Rural Affairs, Wuhan 430070, China; Potato Engineering and Technology Research Center of Hubei Province (HZAU), Wuhan 430070, China; National Key Laboratory for Germplasm Innovation & Utilization of Horticultural Crops, Huazhong Agricultural University (HZAU), Wuhan 430070, China; Key Laboratory of Potato Biology and Biotechnology (HZAU), Ministry of Agriculture and Rural Affairs, Wuhan 430070, China; Potato Engineering and Technology Research Center of Hubei Province (HZAU), Wuhan 430070, China; National Key Laboratory for Germplasm Innovation & Utilization of Horticultural Crops, Huazhong Agricultural University (HZAU), Wuhan 430070, China; Hubei Hongshan Laboratory (HZAU), Wuhan 430070, China; Key Laboratory of Potato Biology and Biotechnology (HZAU), Ministry of Agriculture and Rural Affairs, Wuhan 430070, China; Potato Engineering and Technology Research Center of Hubei Province (HZAU), Wuhan 430070, China

## Abstract

Chloroplasts play a crucial role in essential processes, such as photosynthesis and the synthesis of primary and diverse secondary metabolites. Recent studies have also highlighted their significance linked to phytohormone production in plant immunity, especially SA and JA. Ubiquitination, a key posttranslational modification, usually leads to target protein degradation, which acts as a signal for remodeling the proteome via the induction of protein endocytosis or targeting to other membrane associated systems. Previously, the potato E3 ligase StRFP1 was shown to enhance resistance against *Phytophthora infestans*, but its mechanism remained unclear. Here, we demonstrate that StRFP1 interacted with the dually localized plastid glucose 6-phosphate transporter StGPT1 on the endoplasmic reticulum (ER). Transiently expressed StGPT1-GFP located on the chloroplast and ER in plant cells. Overexpression of *StGPT1* enhances late blight resistance in potato and *Nicotiana benthamiana*, activates immune responses, including ROS bursts and up-regulation of PTI marker genes. The resistance function of StGPT1 seems to be related to its dual localization. Remarkably, StRFP1 ubiquitinates StGPT1 at the ER, possibly due to its merely transient function in peroxisomes, leading to apparent accumulation in chloroplasts. Our findings point to a novel mechanism by which a plant E3 ligase contributes to immunity via interacting with dually targeted GPT1 at the ER of plant cells.

## Introduction

Plants and pathogens have been engaged in an enduring ‘arms race’ over millions of years of co-evolution [1]. To face the challenges of pathogens and a variable environment, plants have developed precise control mechanisms to maintain homeostasis at both the transcriptional and posttranscriptional levels [2]. Among the posttranscriptional modifications (PTMs), the ubiquitin–proteasome system (UPS) stands out as a versatile tool. It attached ubiquitin to target proteins mostly via lysine (K) and rarely via cysteine (C) residues, like in case of peroxisomal import Pex5 receptor recycling [3], regulating their degradation, activity, sorting, and localization [4, 5], which is essential for adapting to the ever-changing environment. In this context, E3 ligases are highlighted due to their targeting of specific substrates [6]. Depending on their distinct structural and functional characteristics, E3 ubiquitin (Ub)-ligase enzymes are generally classified into three subfamilies: the Really Interesting New Gene (RING) and U-box type, the homologous to E6-associated protein C-terminus (HECT) type, and the RING between RING (RBR) type [7]. Some of these subgroups have been identified to play roles in regulating plant immunity-related component turnover, endocytosis, and cytoplasmic transport [8]. For instance, RLCK *Botrytis*-induced kinase 1 (BIK1) is ubiquitinated by AtRHA3A and AtRHA3B, facilitating its endocytosis and activating signaling in BIK1 induced immune response [9]. The ubiquitination of brassinosteroid insensitive 1 (BRI1), a plasma membrane (PM) receptor for brassinosteroids (BRs), is vital for its internalization from the cell surface to the trans-Golgi network/early endosomes (TGN/EEs) [10]. These examples illustrate the diverse functions of E3 ligases in orchestrating vital cellular processes and responses in plants.

Arabidopsis Tóxicos en Levadura (ATL, engl. for ‘toxic in yeast’) is a RING-type plant-specific E3 Ub-ligase subfamily with one or more transmembrane (TM) domains followed by a RING-H2-type domain. Thus far, 80 ATL family genes have been identified in Arabidopsis, 121 in rice, and 96 in grape [11]. Extensive research highlights the pivotal role of ATLs in various aspects of plant growth and development, and ATLs are also implicated in response to biotic and abiotic stresses [12, 13]. For instance, overexpression of *VriATL156* in grapevine leads to strong modulation of the resistance signaling cascade and induces the resistance response [14], and overexpression of *StRFP1* enhances potato resistance against *Phytophthora infestans* [15]. At present, the functional understanding of plant ATLs remains limited, and the substrates of most ATLs remain largely unidentified [16].

Plastids, a set of ancient essential organelles, are widely present in plants and various algae [17]. Among them, chloroplasts stand out for their distinct contribution to photosynthesis and synthesizing vital primary and secondary metabolites [18], and different types of plastids change their status according to developmental processes or environmental cues [19]. During the course of evolution, roughly 98% of the genes of ancestral chloroplasts were either lost or transferred into the cell nucleus, leaving only 100 to 250 genes involved in photosynthesis or linked to metabolite synthesis within chloroplasts [20]. Consequently, chloroplast morphogenesis and function rely on the collaboration of nuclear and chloroplast genomes [21].

In recent years, chloroplasts have emerged as key players in triggering defensive hormonal responses, synthesizing precursors of JA and SA, next to specific reactive oxygen species (ROS) signatures during plant–pathogen interactions [22, 23]. Fascinatingly, chloroplasts create a nexus between the surrounding organelles by clustering around the nucleus or extending thin tubules, known as stromules [24]. This collaboration enables rapid exchange of substances and information, allowing the chloroplasts and the nucleus to jointly regulate the expression of chloroplast and nuclear encoded genes. This insight underlies the idea that stromules facilitate bidirectional movement of proteins and metabolites [25, 26, 27]. In the context of *P. infestans* infection, it was found that plastid clusters with stromules dynamically formed and accumulated at the pathogen interface [28].

The plastidic metabolism is intertwined with that of the surrounding cytoplasmic matrix and other organelles, like the endoplasmic reticulum (ER), facilitated by an array of transporters present on the respective membranes that enable metabolite transport [29, 30]. The plastid phosphate translocator (pPT) family is responsible for metabolite and inorganic phosphate exchange between plastids and the cytosol. Glucose 6-phosphate transporters (GPTs), located on the plastid inner membrane [24, 29], specifically mediate the transport of Glc6P, a crucial substrate for starch and fatty acid synthesis. Additionally, the oxidative pentose phosphate pathway (OPPP) generates pentose–phosphate sugars and NADPH [31], both of which are essential for the assimilation and biosynthesis of fatty acids, as well as other compounds [32, 33]. Intriguingly, NADPH, derived from the irreversible cytosolic OPPP reactions, serves as a substrate for NADPH oxidases, in plants known as respiratory burst oxidase homolog (RBOH), which are responsible for generating reactive oxygen species (ROS) at the plasma membrane/apoplast [34], and may be involved in plant immunity. The GPT subfamily comprises antiporters of partially overlapping substrate preferences, and has been identified in various species, including *Arabidopsis*, maize, pea, soybean, and potato [35, 36].

As reported earlier, StRFP1, an ATL-type E3 ligase, positively regulates host resistance against *P. infestans* [15]. However, our understanding of the molecular mechanism remains limited. In this study, we report the identification of a StRFP1 interacting protein, StGPT1, which is homologous to a dually localized glucose 6-phosphate transporter involved in the plastid oxidative pentose phosphate pathway. We demonstrate that overexpression of StGPT1 enhances potato and tobacco resistance against *P. infestans* and activates various immune responses. Interestingly, StRFP1 ubiquitinated StGPT1 at the ER, which led to the formation of high molecular weight products and disappearance of unprocessed StGPT1 precursors, resulting in accumulation of mature StGPT1 in chloroplasts. This study provides insights on how a plant E3 ligase contributes to immunity by selective interaction with dually targeted GPT1 at the ER during plant–pathogen responses.

## Results

### StGPT1 interacts with StRFP1

StRFP1 is an E3 ligase involved in plant immunity against *P. infestans* [15]. To investigate potential interacting partners, a yeast two-hybrid (Y2H) screen was performed against a DUAL membrane system library generated from *P. infestans*-inoculated potato leaves [37], from which many candidates were identified (Table S2). Through this screening, *StGPT1* (GenBank LOC102578523) emerged as a candidate. *StGPT1* codes for a glucose 6-phosphate transporter, which is primarily associated with plant reproductive development, starch content, and yield [38], but its function related to plant immunity has remained unexplored.

**Figure 1:**
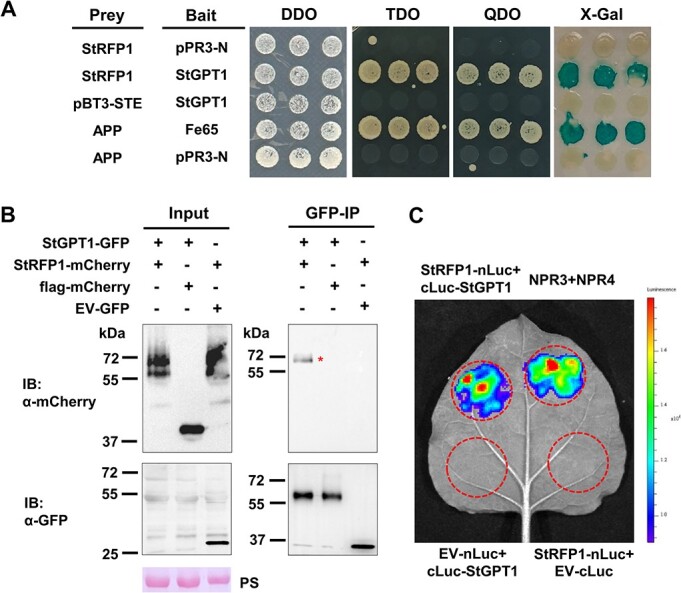
StRFP1 interacts with StGPT1 in yeast and in planta*.*  **A** Yeast cells were plated on YPDA medium with X-gal, SD/-Leu-Trp (DDO), SD/-Leu-Trp-His (TDO), and SD/-Leu-Trp-His-Ade (QDO). **B** Total proteins were extracted, followed by IP with GFP-trap beads. GFP-EV and flag-mCherry were used as negative controls. IB, immunoblotting. + indicates expression of constructs in *N. benthamiana* leaves. Protein size given in kilodalton (kDa), protein loading indicated by Ponceau stain (PS) of Rubisco large subunit. ^*^ indicates target protein band. **C** Luciferase complementation assay (LCA) confirming interaction of StRFP1 and StGPT1. The same leaf was agro-infiltrated with different construct combinations. The fluorescence signal was observed and imaged 48 hours after agro-infiltration. EV, empty vector.

To validate the StGPT1 and StRFP1 interaction, we conducted a DUALmembrane pairwise interaction assay. The interaction between StGPT1 and StRFP1 in yeast was indicated by yeast growth on TDO and QDO plates and an X-gal assay (Figure 1A). To further confirm their interaction, luciferase complementation assay (LCA) and co-immunoprecipitation (Co-IP) were performed in *N. benthamiana* leaves. Co-IP result showed that StRFP1-mCherry could be immunoprecipitated by StGPT1-GFP. Negative control combinations (StRFP1-mCherry + GFP-EV, flag-mCherry + StGPT1-GFP) showed no interactions (Figure 1B, Figure S1A). Furthermore, Myc-StRFP1 was successfully immunoprecipitated by StGPT1-GFP, while Myc-GUS was not (Figure S1B). Upon successful LCA construct expression (Figure S2A), LCA revealed luciferase signals in agro-infiltration sites with Myc-StRFP1-nLuc + HA-cLuc-StGPT1, similar to the positive control (NPR3 + NPR4) (Figure 1C) [39]. These results firmly prove the specific association of StGPT1 with StRFP1 both in yeast and in planta.

It has been reported that the StGPT1 homologous protein AtGPT1 exhibits three forms: precursor, transient, and mature [36, 40]. When StGPT1-GFP was expressed in *N. benthamiana* leaves, three forms were visualized. They are: the precursor (molecular mass about 72 kDa), intermediate (about 65 kDa) and mature form (without cTP) with molecular mass of 60 kDa, respectively (Figure S2B).

### StGPT1 localizes to the chloroplast and ER

**Figure 2:**
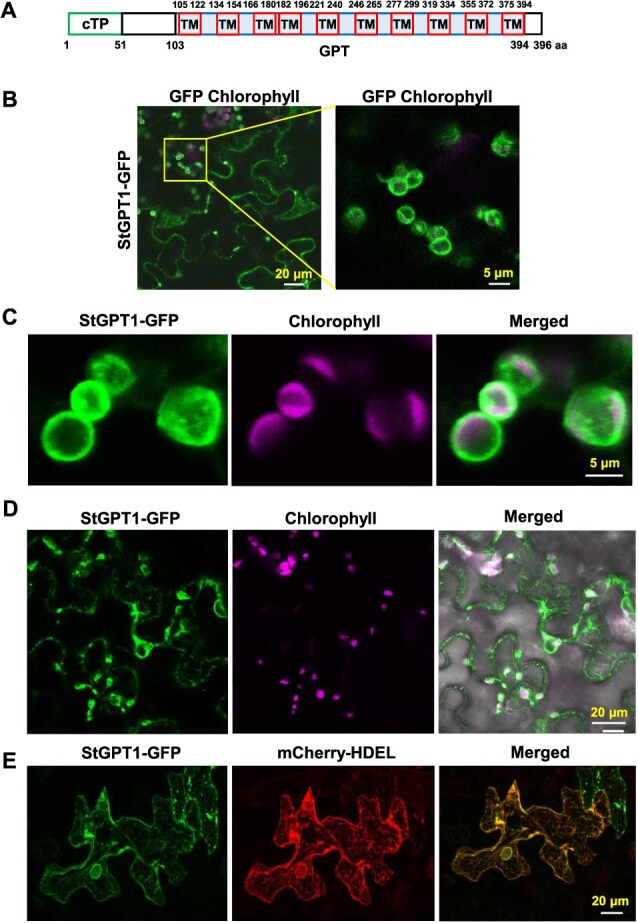
StGPT1 localizes to chloroplast and ER. **A** Schematic of StGPT1, which contains two domains, cTP and GPT. The GPT domain includes ten TM motifs. **B** Confocal images showing StGPT1-GFP localized to chloroplast and ER. Amplified image (right) shows StGPT1-GFP co-localized with chloroplast. **C** Confocal images showing StGPT1-GFP localized to chloroplast. **D** Confocal images showing StGPT1-GFP localized to chloroplast and ER in same cells. ‘Merged’ one includes the bright field image. **E** Co-expression of StGPT1-GFP with an ER marker (mCherry-HDEL). Constructs were agro-infiltrated in *N. benthamiana* leaves. In **B**-**E**, each image represents one section confocal image.

StGPT1 contains a chloroplast transit peptide (cTP) that may guide it to chloroplasts or plastids, along with a GPT family-specific domain comprising ten transmembrane domains [41] (Figure 2A, Figure S3A). *Solanum tuberosum* contains two GPTs, StGPT1 and StGPT2, with 78.69% amino acids sequence similarity (Figure S3B). As Baune et al. [40] reported, the GPT family exhibits evolutionary conservation, with the GPT functional domain displaying minimal differences; variations primarily occur in the N-terminus, including the cTP (Figure S3).

AtGPT1 is a dually targeted protein. AtGPT1-GFP localized to plastids, but also to the endoplasmic reticulum (ER) and around peroxisomes [40]. When StGPT1-GFP was transiently expressed in the leaves of *N. benthamiana*, confocal microscopy illustrated that StGPT1-GFP localized to both chloroplasts, aligned with the auto-fluorescence of chloroplasts, as well as to the ER (Figure 2B, C, D), as indicated by the overlap of green fluorescence with the marked ER visualized by mCherry-HDEL (Figure 2E). We further investigated whether StGPT1 co-localized with AtGPT1. StGPT1-GFP and AtGPT1-mCherry were agro-infiltrated into *N. benthamiana* leaves. A strong coincident fluorescence pattern was observed between StGPT1 and AtGPT1 specifically on the ER (Figure S4B). These observations confirm that StGPT1 localizes to both the chloroplasts and ER when it was transiently expressed in *N. benthamiana* leaves.

### Over-expression of StGPT1 reduces *P. infestans* colonization of host plants

To determine whether *StGPT1* responds to *P. infestans* infection, the expression levels of *StGPT1* and *StWRKY7* (as a positive control) were examined in potato leaves at 0, 12, 24, 36, 48, and 60 hours post *P. infestans* inoculation. The results demonstrated a significant increase of *StGPT1* during the infection, along with *StWRKY7*, which was strongly induced by *P. infestans*, though less intensely than *StWRKY7* (Figure S5).

To investigate the role of StGPT1 in late blight resistance, stable transgenic *N. benthamiana* lines with construct *35S::StGPT1-GFP* were generated and virus-induced gene silencing (VIGS) assay was applied. There was no phenotypic difference between wild-type *N. benthamiana* and OE transgenic lines (Figure S6A). Western blot showed that a 60-kDa form of StGPT1 was detected in transgenic lines (Figure S6B). Fluorescence of StGPT1-GFP was only observed in chloroplasts in stable transgenic tobacco lines (Figure S6C). Similarly, it has been shown that OsGPT1-GFP (under control of the double 35S promoter) was exclusively localized to the plastids in rice protoplasts [42].

**Figure 3:**
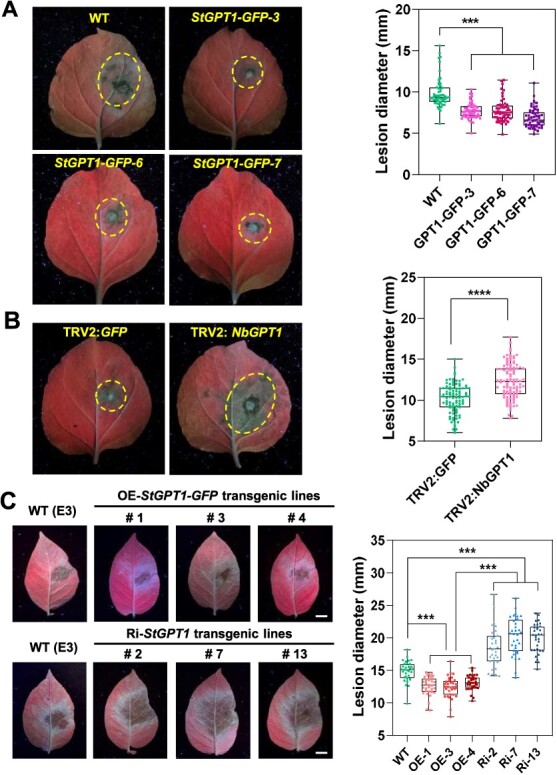
StGPT1 positively regulates *P. infestans* resistance. **A** Representative image showing disease lesion diameters on overexpression transgenic *StGPT1 N. benthamiana* lines at 5 days postinoculation (dpi) with *P. infestans* isolate 88069, wild-type *N. benthamiana* as negative control. One-way ANOVA was used for statistics analysis (^****^  *p* < 0.0001, three independent repeats, n = 64). **B** Silencing *NbGPT1* reduces late blight resistance in *N. benthamiana* (^****^  *p* < 0.0001, three independent repeats, *t*-test, n = 100). **C** Representative images showing disease lesion diameters on potato OE- and Ri- lines of *StGPT1* at 5 dpi by isolate HB0914-2, wild-type as a negative control. Statistics of disease lesion diameters. One-way ANOVA was used for statistical analysis (^***^  *p* < 0.001, three independent repeats, n = 33). Error bars indicate ± SE.

The leaves of transgenic StGPT1-OE *N. benthamiana* lines (3–4 weeks) were subsequently inoculated with *P. infestans* isolate 88069 and found to have significantly lower pathogen colonization, as measured by smaller disease lesion diameters compared with wild-type control (Figure 3A). Consistent with this, silencing of *NbGPT1* in *N. benthamiana* by VIGS led to increased susceptibility to *P. infestans* and stunted growth (Figure S6D, Figure 3B), which indicates that *NbGPT1* counteracts *P. infestans* colonization and may be involved in *N. benthamiana* plant growth. Constructs of *35S::StGPT1-GFP* and RNA-interfering (RNAi) pHellsgate8-*StGPT1* were transformed into potato cultivar, 'E-potato 3' (E3) to obtain stable transgenic potato lines. Overexpression lines exhibited 10 to 30 times higher transcript levels of *StGPT1*, yet displayed no significant phenotypic changes compared to wild-type (Figure S6E). Conversely, RNAi lines showed only 30% to 60% of the wild-type transcript level and exhibited retarded growth, notably in Ri-7 (Figure S6F). Subsequent inoculation with *P. infestans* isolate HB09-14-2 showed that significantly smaller disease lesions were observed in overexpression lines (OE-1, OE-3, and OE-4), while RNAi lines (Ri-2, Ri-7, and Ri-13) showed larger lesions compared to the control wild-type (Figure 3C). These findings collectively underscore the positive role of StGPT1 against *P. infestans* infection and its potential role in plant growth and development.

### StGPT1 activates plant immunity responses

Besides being linked to primary metabolism and the synthesis of phytohormones and many secondary metabolites, chloroplasts play a pivotal role in orchestrating plant defense, including participating in retrograde signaling and reactive oxygen species (ROS) production, which is important for successfully activating the immune responses and limit the pathogens invasion [23].

**Figure 4:**
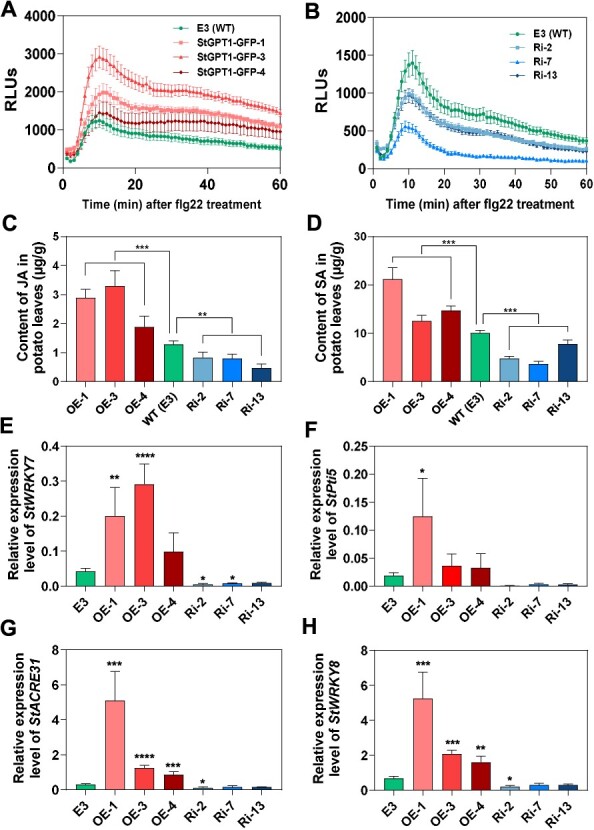
Overexpression of *StGPT1* enhances plant immune response. **A-B** flg22-induced ROS production in *StGPT1*-OE and *StGPT1*-RNAi transgenic potato lines, respectively. Leaves were treated with 10 μM flg22 before measuring ROS production. RULs, relative luminescence units. **C-D** JA or SA content in leaves of *StGPT1* transgenic potato lines (^**^  *p* < 0.01, ^***^  *p* < 0.001; three to four biological repeats). **E-H** Relative expression of PTI marker genes *StWRKY7*, *StWRKY8*, *StACRE31*, and *StPti5*, respectively, in *StGPT1* transgenic potato lines (^*^  *p* < 0.1, ^**^  *p* < 0.01, ^***^  *p* < 0.001, ^****^  *p* < 0.0001, three biological repeats). Error bars indicate ± SE.

To investigate whether StGPT1 modulates the chloroplast-mediated immune response, we evaluated the production of ROS, as well as the levels of chloroplast-associated hormones, jasmonic acid (JA) and salicylic acid (SA), in *StGPT1*-OE and RNAi potato lines. The results showed that following treatment with flg22, elevated and robust ROS production was observed in the *StGPT1*-OE lines (Figure 4A), while a less pronounced ROS response was recorded in the *StGPT1*-RNAi lines (Figure 4B). Similarly, the contents of both SA and JA were also upregulated in the OE- lines and downregulated in the RNAi lines (Figure 4C, D). These results highlight the role of functional StGPT1 in immune response activation. In addition, we examined the expression levels of marker genes involved in PAMP-triggered immunity (PTI) in *StGPT1* transgenic potato lines. *StACRE31*, *StPti5*, *StWRKY7*, and *StWRKY8* were all upregulated in *StGPT1*-OE lines and decreased in *StGPT1-*RNAi lines (Figure 4E–H). The above results demonstrate that enhanced expression of *StGPT1* in plants contributes to the augmented immune response.

### Positive function of StGPT1 regulating resistance against *P. infestans* relies on its chloroplast localization

**Figure 5:**
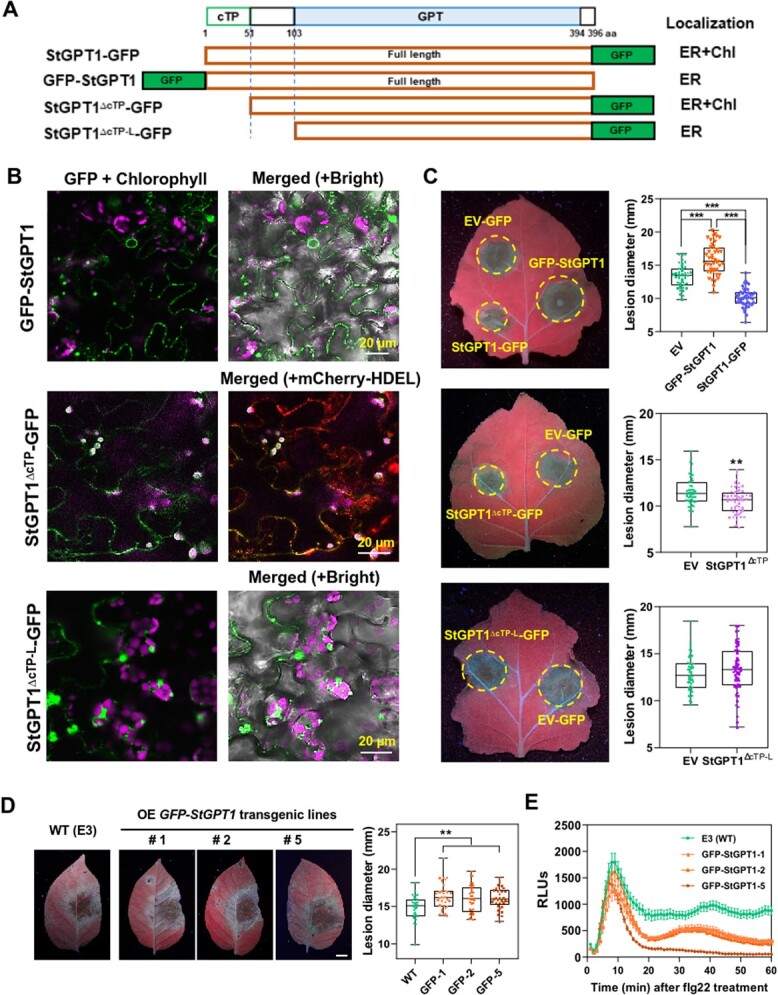
Positive regulation of StGPT1 in late blight resistance relies on its chloroplast localization. **A** Schematic of StGPT1 mutants. **B** Localization of GFP labeled StGPT1 mutants: one optical section (left), 10 optical sections (right). GFP fluorescence indicates mutants, pink indicates spontaneous chloroplast fluorescence, red indicates mCherry-HDEL (ER maker). **C** Representative images showing diameters of disease lesions on *N. benthamiana* leaves at 5 dpi with *P. infestans* isolate 88069 (^**^  *p* < 0.01, ^***^  *p* < 0.001, three independent repeats). One-way ANOVA was used for statistical analysis of *GFP-StGPT1*, n = 43. *t* - test was used for statistical analysis of StGPT1^ΔcTP^-GFP and StGPT1^ΔcTP-L^-GFP, n = 58 and 56, respectively. **D** Representative images showing diameters of disease lesions on transgenic *GFP-StGPT1* potato leaves at 5 dpi with *P. infestans* isolate HB0914-2, wild-type as negative control. One-way ANOVA was used for statistical analysis (^**^  *p* < 0.01, three independent repeats, n = 33). **E** flg22-induced ROS production in *GFP-StGPT1* transgenic potato lines. Leaves were treated with 10 μM flg22 before measurement. RULs, relative luminescence units. EV, empty vector.

StGPT1 dually localized to the ER and chloroplasts when it was transiently expressed in *N. benthamiana* (Figure 2). StGPT1 contains a cTP that ultimately guides it to the chloroplasts, and *StGPT1*-OE transgenic plants showed enhanced resistance against *P. infestans* (Figure 3; Figure S6C). In order to further explore where StGPT1 exerts its resistance function, GFP-StGPT1 (GFP was tagged to the N terminal of StGPT1 with interfered cTP to disturb its chloroplast localization) and StGPT1^ΔcTP^-GFP with a deleted cTP domain were constructed (Figure 5A). Confocal images showed that GFP-StGPT1 mainly resided on the ER, while StGPT1^ΔcTP^-GFP displayed both ER signal and partial chloroplast localization (Figure 5B). Then, transient expression and *P. infestans* inoculation were performed to test their function. Leaves expressing GFP-StGPT1 were more susceptible to *P. infestans* 88069 than the negative control EV-GFP; conversely, StGPT1-GFP maintained its resistance. Intriguingly, leaf sites expressing StGPT1^ΔcTP^-GFP also displayed resistance compared to the control (Figure 5C). This suggests that cTP deletion may not be complete in the disruption of chloroplast localization and its resistant function. As a proof of concept, we introduced StGPT1^ΔcTP-L^-GFP with 1 to 103 aa deleted mutant (Figure 5A), which only localized to the ER and resulted in similar disease lesions as the control (Figure 5B, C).

Potato has two StGPTs, StGPT1 and StGPT2 (Figure S3A). It has been shown that AtGPT2 exclusively located to the plastids [40]. We confirmed that the chloroplast localization of StGPT2 is similar to that in *Arabidopsis* (Figure S7A). To further investigate whether the chloroplast localization is essential for StGPT1 resistant function, cTP of the StGPT2 was fused to StGPT1 to replace its native cTP. Confocal microscopy revealed that StGPT1^cTP-StGPT2^-GFP exhibited the exclusive chloroplast localization (Figure S7B), and it plays a significant resistance role against *P. infestans* (Figure S7C). In summary, results above indicate that the chloroplast localization is important for StGPT1 to fulfill its resistance function in plants. All constructs used for function analysis were successfully expressed (Figure S8).

To provide more direct evidence, transgenic potato lines overexpressing *GFP-StGPT1* were generated. The relative expression level of *StGPT1* is shown in Figure S6G, and these plants also exhibited growth inhibition compared to the wild-type. Strikingly, the potato *GFP-StGPT1* OE transgenic lines displayed increased susceptibility to *P. infestans* and reduced ROS production compared to the wild-type (Figure 5D, E). These findings further solidify the role of chloroplast-localized StGPT1 in positively regulating late blight resistance.

### StRFP1 and StGPT1 co-localize on the ER and synergistically enhance late blight resistance

**Figure 6:**
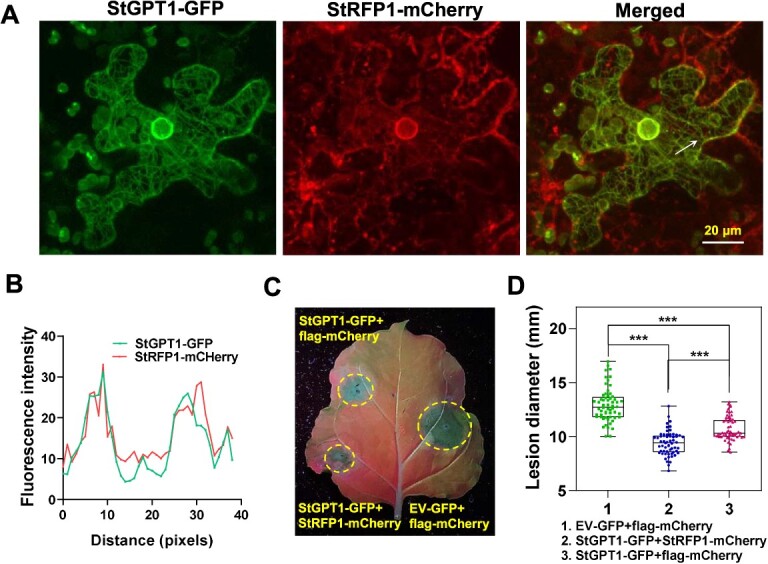
StRFP1 and StGPT1 co-localize on ER and synergically enhance *P. infestans* resistance. **A** StGPT1 co-localized with StRFP1 mainly on ER (the bright circle is the center represents the peri-nuclear ER). Bar is 20 μm. **B** Plots of profiles indicated by arrows (right image) show fluorescence intensity of StGPT1 (green line) and StRFP1 (red line). **C** Representative image showing diameters of disease lesions on *N. benthamiana* leaves at 5 dpi with *P. infestans* isolate 88069. **D** Plot graph showing differences in lesion diameters. One-way ANOVA was used for statistical analysis, ^***^  *p* < 0.001, three independent repeats, n = 60. Error bars indicate ± SE. EV, empty vector.

StGPT1 was located in the chloroplasts and ER (Figure 2). To investigate where their interaction occurred, StGPT1-GFP and StRFP1-mCherry were transiently co-expressed in *N. benthamiana* leaves. Confocal images show that they were predominantly co-localized on the ER, and the network structure of the ER is very clearly observed (Figure 6A, B; Figure S9A). It is worth noting that StRFP1 is known to be associated with the membrane system and endocytic trafficking, and was not previously observed to be localized on the ER [15, 43]. StRFP1-GFP was co-expressed with mCherry-HDEL, an ER marker, in *N. benthamiana*. Confocal images show that StRFP1 wrapped around the ER as puncta without actual co-localizing with mCherry-HDEL (Figure S9B), which may reflect endosomal localization when StGPT1 is not co-expressed. StRFP1 belongs to the ATL protein family. To confirm the specific interaction between StGPT1 and StRFP1, *Arabidopsis* ATL1, an ATL family E3 ligase that localizes to endosomes and plasma membrane [44], similar to the subcellular localization of StRFP1, was applied to test whether it interacts with StGPT1. As shown in Figure S9C, ER localization of StGPT1 was clearly observed, while ATL1 appeared to surround the ER without changing the membrane system localization or co-localizing with StGPT1. Co-IP assay showed that StGPT1 did not interact with AtATL1 or ER protein HDEL (Figure S9D), demonstrating that StGPT1 specifically interacted with StRFP1 on the ER.

Given that both StGPT1 and StRFP1 positively regulate late blight resistance, we wondered whether they would show a synergistic effect on *P. infestans* resistance. StGPT1 and StRFP1 were co-expressed in *N. benthamiana*. Subsequent *P. infestans* inoculation assay showed that the sites co-expressing StGPT1 and StRFP1 had significantly smaller disease lesions compared to the sites expressing StGPT1 or flag-mCherry control (Figure 6C, D), indicating that they could synergistically increase *P. infestans* resistance.

### StRFP1 ubiquitinates StGPT1 and promotes StGPT1 accumulation in the chloroplast

Previous results indicated that the positive regulatory role of StRFP1 in plant immunity relies on its E3 ligase function [15]. The interaction between StGPT1 and StRFP1 and their synergistic role in enhanced *P. infestans* resistance strongly suggests that StGPT1 is not a substrate of StRFP1 for immediate degradation. In *StGPT1-GFP* OE transgenic *N. benthamiana* leaves, only a mature StGPT1-GFP band (cTP was cleaved, about 60 kDa) was detected by immunoblot analysis and the green fluorescence was only detected in the chloroplasts (Figure S6B, C).

**Figure 7:**
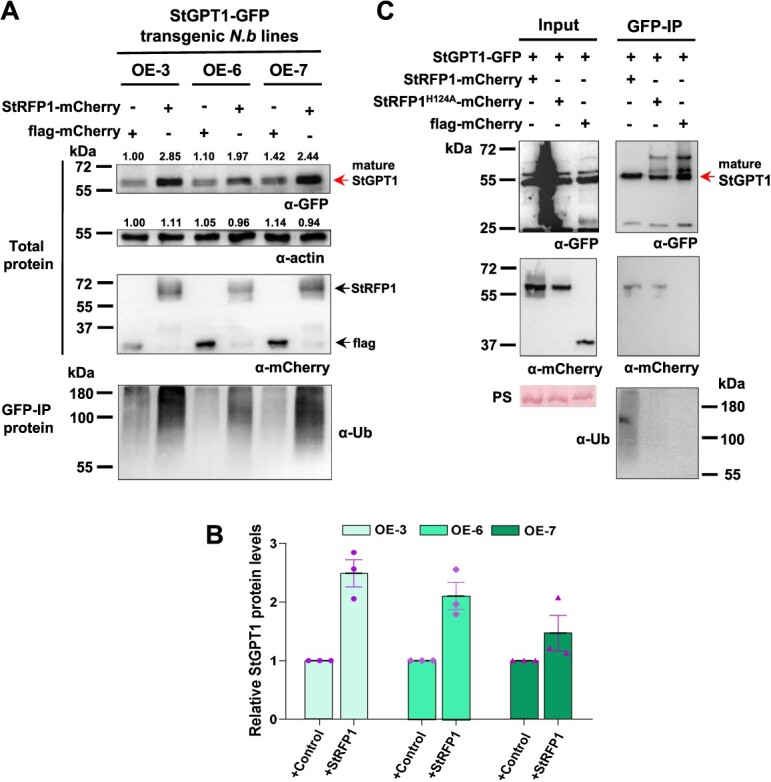
StRFP1 ubiquitinates StGPT1 and promotes it mature chloroplast form accumulation. **A** Western blot shows that the mature form of StGPT1 accumulated and ubiquitination level increased in the presence of StRFP1. Expression of constructs is indicated by +. Protein size is given in kilodalton (kDa). GFP beads trapped proteins from *StGPT1* transgenic *N. benthamiana* leaves in which StRFP1- or flag-mCherry was transiently expressed. **B** Relative StGPT1 protein levels in leaves of three *StGPT1-GFP* transgenic *N. benthamiana* lines upon transient expression of StRFP1- or flag-mCherry. The StGPT1 protein level was normalized to actin. Data were collected from A and Figure S10. **C** Co-IP assay shows that StRFP1 or StRFP1^H124A^ interacts with StGPT1. Total proteins were extracted, followed by IP with GFP-trap beads. Flag-mCherry was used as negative control. + indicates expression of constructs in *N. benthamiana* leaves. Protein size given in kilodalton (kDa), protein loading indicated by Ponceau stain (PS) of Rubisco large subunit. Red arrow indicates mature StGPT1-GFP band.

To further analyze the influence of the E3 ligase StRFP1 on StGPT1, StRFP1 was transiently expressed in *StGPT1-*OE transgenic *N. benthamiana* leaves. Anti-Ub blotting indicated strong ubiquitin–conjugate formation with StGTP1. When co-expressed with flag-mCherry, there was also weak ubiquitination of StGPT1, suggesting the possibility of StGPT1 ubiquitination by other E3 ligases (Figure 7A, Figure S10). However, a marked elevation of StGPT1-GFP protein levels were observed when StRFP1 was transiently expressed in StGPT1-OE transgenic *N. benthamiana* leaves (Figure 7A), that was further confirmed by statistical analysis of the quantitative data of StGPT1-GFP protein levels from three independent experiments (Figure 7B). Because StGPT1-GFP band (about 60 kDa) represents mature form in the chloroplasts, we speculated that the interaction of StGPT1 with StRFP1 promotes its accumulation in the chloroplasts. To test whether the promotion is related to the E3 ligase of the StRFP1, StRFP1^H124A^, a mutant without E3 ligase activity [15] was used to further determine whether the ubiquitination of StGPT1 is necessary for its accumulation in the chloroplasts. Western blot showed that, in GFP-IP proteins, the mature StGPT1-GFP protein band is weak when StGPT1-GFP was co-expressed with StRFP1^H124A^ compared to with the intact StRFP1 (Figure 7C; Figure S11). StGPT1 also interacts with StRFP1^H124A^, but it was not ubiquitinated by it. Meanwhile, StGPT1 maintains its three distinct isoforms when it was co-expressed with StRFP1^H124A^ or with control flag-mCherry, which is very different from its behavior when co-expressed with the intact StRFP1. Based on these findings, it is obvious that ubiquitination of the StGPT1 precursors by StRFP1 contributes to accumulation of the mature StGPT1 version in chloroplasts.

## Discussion

In this paper, we report that StRFP1, an E3 ligase, collaborates with StGPT1, a dually targeted protein that is localized in chloroplasts and the endoplasmic reticulum (ER), to synergistically regulate plant resistance against *P. infestans*. Notably, StRFP1 increased the ubiquitination level of StGPT1 precursors, yet did not lead to detectable degradation. Nevertheless, StRFP1-StGPT1 co-expression enhanced the abundance of the mature StGPT1 form (Figure 7). StGPT1 was first reported to be involved in plant immunity against *P. infestans* in *N. benthamiana* and potato (Figure 3). This study highlights the role of StGPT1, a dually targeted protein, in plant immunity, revealing specific interaction with an E3 ligase in a defense mechanism against *P. infestans*.

GPT is extremely crucial for plastid carbon uptake in most non-green tissues, and loss of GPT leads to disruption of the OPPP cycle, which, in turn, affects the fatty acid or carbohydrate biosynthesis in plastids, including chloroplasts [33, 45]. Almost all dicotyledons possess both GPT1 and GPT2, indicating their important roles [40, 46, 47]. Although GPT1 and GPT2 are highly similar in their amino acids, they have notable functional differences. In *Arabidopsis*, deletion of GPT1 proved to be lethal, but GPT2 disruption had a minimal effect on plant growth under greenhouse conditions [31]. In this study, we confirmed that interference of GPT1 in potato and *N. benthamiana* inhibited plant growth (Figure S6F). *GPT1* is ubiquitously expressed, including in leaves, whereas *GPT2* expression is generally low and mainly limited to heterotrophic tissues, except for stress conditions, like high light in *Arabidopsis* [48] or *P. infestans* infection [49]. Thus, likely GPT1, not GPT2, provides sugar phosphates to stabilize the Calvin cycle in chloroplasts [40, 50]. GPT1 may transport G6P from the cytoplasm into plastids as a substrate to provide NADPH for fatty acid synthesis via the OPPP pathway [51, 52]. In addition, GPT2 only localizes to plastids, while GPT1 resides on both the plastids and the ER [40]. Here, we observed that StGPT1, similar to AtGPT1, localized to both the chloroplasts and the ER (Figure 2; Figure S4), while StGPT2 only localized to the chloroplasts (Figure S7A).

Remarkably, we noticed a gradual up-regulation of *StGPT1* during *P. infestans* infection (Figure S5), which is consistent with elevated mRNA expression of *AtGPT1* under this condition [49] that may be related to the reinforcement of chloroplast-based reactions related to immunity. Chloroplasts are not only pivotal for photosynthesis and primary metabolism but also serve as sites for the synthesis of plant hormones, including precursors of jasmonic acid (JA) and salicylic acid (SA), as well as abscisic acid (ABA) derived from the plastid. These hormones and their crosstalk play crucial roles in orchestrating plant defenses against various stressors, including immune responses [53]. In our study, we observed that the overexpression of *StGPT1* enhanced the resistance against late blight in transgenic potato, accompanied by an increased production of ROS and the accumulation of JA and SA, along with the up-regulation of PTI marker genes (Figure 4). These findings suggest that StGPT1 contributes to the late blight resistance in potato.

Ubiquitination is generally considered to regulate protein degradation. It also plays an important regulatory role in protein activity and subcellular localization, which may also lead to substrate accumulation [54]. IPI1 (IPA1 INTERACTING PROTEIN 1), a rice RING-finger-type E3, promotes the turnover of IPA1 (IDEAL PLANT ARCHITECTURE 1) in panicles, while IPI1 stabilizes IPA1 and increases protein abundance in shoot apexes [55]. In this study, we found that StRFP1 interacted with, and ubiquitinateed StGPT1, but did not lead to its obvious turnover, yet enhanced the abundance of mature StGPT1 (Figures 7). Mature StGPT1 is usually located in chloroplasts. We show that the chloroplast localization is necessary for the StGPT1 to enhance late blight resistance (Figure 5), and StRFP1-StGPT1 interaction had a cumulative impact on the resistance against *P. infestans* (Figures 6C, D). A possible explanation would be that ubiquitination of the StGPT1 by StRFP1 at the cytoplasmic face of the ER promotes appearance of the mature form in chloroplasts. Interestingly, although mutant StRFP1^H124A^ lacks E3 ligase activity, the mutant protein still interacted with StGPT1, yet lost the ability to enhance the abundance of mature StGPT1 (Figures 7; Figure S11). This indicated that StGPT1 precursor ubiquitination by StRFP1 at the ER is necessary for accumulation of the mature form in chloroplasts. In fact, StGPT1^∆cTP^ and StGPT1^cTP-StGPT2^ did not interact with StRFP1 anymore (Figure S11), indicating that the N-terminal chloroplast transit peptide (cTP) of StGPT1 is important for the interaction of StGPT1 with StRFP1. Because the cTP was cleaved in mature StGPT1 within the chloroplasts, it is reasonable that the association of StRFP1 and StGPT1 occurred in the cytosol, likely on the ER. Different ubiquitin chain modulates distinct protein destination in cells [56]. Ubiquitin chains linked via K11 or K48 to target proteins always lead to degradation by the proteasomal system, whereas K63-linked ubiquitination is involved in several processes, including the dynamics of organelles and endomembrane compartments (vesicular trafficking, endocytosis, fission of mitochondria), and the organization of the nucleus (DNA topology, nuclear envelope, microtubules, and transcription) [56, 57, 58]. However, what happens during interaction between StGPT1 and StRFP1 on the ER, what kind of ubiquitin chain is linked to StGPT1, and the connection with StGPT1 import into chloroplasts that was promoted by ubiquitination of its precursors remains to be further explored. Besides StGPT1, StRFP1 potentially interacts with other proteins (Supplementary Table S2) that probably involved in establishing enhanced resistance to *P. infestans*. Additional studies are required to validate that.

In summary, this study reveals the role of StGPT1 in disease resistance and sheds light on the intricate ubiquitin signaling and chloroplast protein trafficking in plant cells. It also demonstrates a novel dimension for our understanding of how an E3 ligase interacts specifically with the transit peptide of dually targeted GPT1, but not its close homolog GPT2, to regulate plant immunity.

## Materials and methods

### Plant materials and growth conditions

*Nicotiana benthamiana* plants were grown under long-day conditions of 16/8 hours light/dark at 22°C and 40% to 60% humidity. Then, 3- to 4-week-old plants were used for transient expression assays. Chinese potato cultivar ‘E-potato-3’ (E3) was used to get stable transgenic lines of *StGPT1* and *GFP-StGPT1*. The transgenic potato lines were planted in the greenhouse in a natural environment. Six-week-old potato plants were used for *P. infestans* infection assays. Three experimental replicates were conducted for inoculation. At least 10 leaves were collected from three plants per line for each replicate.

### Plasmid constructs

*StRFP1* [15] and *StGPT1* (LOC102578523) and its mutants *StGPT1^ΔcTP^* and *StGPT1^ΔcTP-L^* were cloned into pH7LIC with a C-terminal GFP tag, C-terminal Myc tag, and N-terminal GFP tag, respectively. StRFP1 and *AtGPT1* (AT5G54800) were fused with a C-terminal mCherry tag in pHellsgate8. All these constructs are controlled by the cauliflower mosaic virus (CaMV) 35S promoter. The vectors containing StGPT1 and GFP-StGPT1 were used to generate transgenic potato lines. RNAi construct with 200 to 300 bp of *StGPT1* specific sequence (Supplementary Table S1) was cloned into the pHellsgate8 to generate pHellsgate8-StGPT1 for RNAi. Construct with 150 bp of *NbGPT1* specific sequence (Supplementary Table S1) was cloned into the TRV2 to apply in virus-induced gene silencing assay.

For yeast two-hybrid (Y2H) assay, the full-length coding sequences of prey vector *StRFP1* was cloned into pBT3-STE, and the bait vector *StGPT1* was cloned into pPR3-N. For split luciferase complementation assay (LCA), *StRFP1* and *StGPT1* were cloned into pCAMBIA1300-nLuc/cLuc, with 3× Myc or 3× HA added to the N-terminus of the target genes to produce Myc-StRFP1-nLuc and HA-cLuc-StGPT1. All vectors were produced by using a ClonExpress® II One Step Cloning Kit (Vazyme Cat.# C112-02). Primer sequences used in this research are listed in Supplementary Table S1.

### *Agrobacterium*-mediated transient expression assay

For confocal, Western blot (WB), immunoprecipitation (IP), or co-immunoprecipitation (Co-IP) assay and LCA, agrobacterium-mediated transient expression was performed in *N. benthamiana*. For this process, 3- to 4-week-old *N. benthamiana* plants were infiltrated with *Agrobacterium tumefaciens* strain GV3101 containing constructs with the addition of gene silencing suppressor P19. After overnight culture, the agrobacteria cells were centrifuged at 4000 g for 10 minutes, and resuspended with an appropriate concentration of MMA (10 mM MES, 10 mM MgCl_2_, 200 μM acetosyringone). Plant leaf materials were sampled 48 hours after infiltration.

### Potato and *N. benthamiana* transformation

Overexpression (OE) constructs *35S::StGPT1-GFP* and *35S::GFP-StGPT1* and RNA-interference construct RNAi-*StGPT1* were individually introduced into *A. tumefaciens* strain GV3101. Then, they were used to transform Chinese potato cultivar ‘E3’, as described by Guo *et al.* [37].

Construct *35S::StGPT1-GFP* was introduced into *A. tumefaciens* strain GV3101 and used to transform *N. benthamiana* leaf discs, as described by Zhou *et al.* [59].

### Virus-induced gene silencing (VIGS)

A 150-bp *NbGPT1*-specific fragment cloned from *N. benthamiana* cDNA (shown in Supporting Information Table S1) was cloned into TRV vector and a TRV construct expressing a fragment of GFP was used as control. The VIGS assay was described by Qi *et al.* [60].

### Quantitative real-time PCR

Potato leaf samples were taken from individual plants and sampled to detect gene expression levels. Three leaves were snap-frozen in liquid nitrogen. Total RNA was extracted from the samples using a Plant Total RNA Kit (ZOMANBIO Cat.# ZP405-1). cDNA was produced with a Reverse Transcriptase Kit (All-In-One 5 × RT MasterMix, Applied Biological Materials, Cat.# G592) and used for quantitative real-time PCR assays as previously described [60]. qRT-PCR was performed on an ABI7300 PCR machine (Applied Biosystems) using qPCR MasterMix (abm 2× qPCR MasterMix, Cat.# G891). Gene expression levels were calculated using a comparative Ct method [61]. qRT-PCR primers are listed in Supplementary Table S1.

### *P. infestans* inoculation assay

*P. infestans* was grown on rye–sucrose–agar medium at 19°C for 2 weeks in the dark. The sporangia were gently washed with ddH_2_O, and the sporangia number was adjusted to 120,000 sporangia/mL. Isolate 88069 was used to inoculate *N. benthamiana* leaves and isolate HB09-14-2 was used to inoculate potato ‘E3’ lines. Lesion diameters were measured at 5 to 7 days postinoculation (dpi).

### Yeast two-hybrid assay

StRFP1 was cloned into pBT3-STE as a bait vector to select the possible interacting proteins against a DUALmembrane system library with cDNAs generated from potato leaf samples inoculated with *P. infestans,* as described by Guo *et al.* [37].

### Split luciferase complementation assay

The combined constructs were co-expressed in *N. benthamiana*, and leaf samples were collected 48 hours after agro-infiltration. Then, 50 mM of luciferin was spread evenly onto the leaves, which were kept in the dark for 15 minutes, then the fluorescence was detected, and images were captured using the NightSHADE LB 985 In Vivo Plant Imaging System.

### Flg22 treatment and ROS test

First, 1 mg of synthetic 22 amino acid flg22 peptide (QRLSSGLRINSAKDDAAGLAIS) was dissolved in 20 μL DMSO. Leaf discs 3 mm in diameter were taken from 4-week-old transgenic potato leaves and kept in 200 μL ddH_2_O in a 96-well plate overnight. After 16 hours, the water was removed and 100 μL reaction solution (1 μM flg22, 0.2 mg/mL HRP, 1 mM luminol) was added, and the sample was quickly placed in an ELISA microplate reader (Tecan Spark, SA) for 60 minutes. Analysis of reactive oxygen species referred to the relevant literature [62]. Luminescence data were analyzed as described by Li *et al.* [62].

### Western blot analysis

Total protein was extracted from *N. benthamiana* with strong-RIPA extraction buffer (150 mM NaCl, 1.0% Triton X-100, 0.5% sodium deoxycholate, 0.1% SDS, 50 mM Tris, pH 8.0), EDTA-free Protease Inhibitor Cocktail Tablet (Roche), 10 mM β-mercaptoethanol, and 0.1 mM PMSF. Each 100 mg of total protein was in 400 μL extraction buffer. The extracts were centrifuged at 13,000×*g* at 4°C for 10 minutes three times, and then heated at 95°C for 10 minutes with 2× SDS loading buffer supplemented with 200 mM dithiothreitol (DTT). SDS-PAGE was used to separate the samples, and gel transfer was performed with PVDF membranes (Whatman) and confirmed by Ponceau staining (Servicebio, Cat.# G2011). Blots were immunodetected with corresponding antibodies (anti-Ub, Enzo, Cat.# ADI-SPA-203-0025; anti-mCherry, EnoGene, Cat.# E12-010-3; anti-GFP and anti-Myc, Bioyears; anti-Actin, Abbkine, Cat.# ABL1050, 1:4000). Proteins were indicated by 180 kDa Prestained Protein Marker (Yeasen, Cat.# 20350). To detect the stability of StGPT1 with or without StRFP1 in transgenic tobacco lines, one leaf was divided into left and right parts, for the expression of StRFP1-mCherry or flag-mCherry. After 48 hours, total protein was extracted with strong-RIPA lysis buffer.

### Immunoprecipitation (IP) or Co-IP assay

Total protein was extracted with extraction buffer (50 mM Tris-HCl, pH 7.5, 150 mM NaCl, 10% glycerol, 0.1% Triton X-100, EDTA-free Protease Inhibitor Cocktail Tablet (Roche), and 0.1 mM PMSF) and subsequently incubated with 25 μL anti-GFP agarose beads (Alpha Biosciences, Cat.# KTSM1301) in a rocking platform at low speed at 4°C for about 6 hours. After incubation, agarose beads were washed five or more times with 800 μL extraction buffer, and then heated at 95°C for 10 minutes in 2× SDS loading buffer supplemented with 200 mM DTT.

### Confocal observation

Forty-eight hours after agro-infiltration in *N. benthamiana* leaves, the fluorescence signals were observed and imaged using a confocal laser scanning microscope (Leica TCS-SPE, Germany) according to the manufacturer's instructions. GFP was observed at an excitation wavelength of 488 nm and emission wavelengths of 496 to 533 nm, and mCherry was observed at an excitation wavelength of 568 nm and emission wavelengths of 568 to 610 nm. Images were processed and quantified by ImageJ.

### Statistical analysis

All data analyses were performed using one-way ANOVA or Student's *t* test and pairwise or multiple comparisons with GraphPad Prism 8.0 software (GraphPad Software Inc.). The error bars in the figures and all values shown are means ± SE of three or more replicates.

## Supplementary Material

Web_Material_uhae241

## Data Availability

The data underlying this article are available in the article and in its online supplementary data.
