# Supplementary material for: Accumulation of dually targeted StGPT1 in chloroplasts mediated by StRFP1, an E3 ubiquitin ligase, enhances plant immunity
Source: Hortic Res. 2024 Aug 30;11(11):uhae241. doi: 10.1093/hr/uhae241 (PMC11540758; doi:10.1093/hr/uhae241)
Supplement: Web_Material_uhae241 [file web_material_uhae241.zip › Supplimental Information.pdf]

Supplemental Information

Supplemental Figures 1-11

Supplemental Tables 1-2

Article Title: Accumulation of StGPT1 in chloroplasts promoted by StRFP1, an E3 ubiquitin ligase, enhances plant immunity

Xintong Wu[1,2,3,4], Xiaoshuang Zhou[1,3,4], Tianyu Lin [1,3,4], Zhe Zhang[1,3,4], Xinya Wu[1,3,4], Yonglin Zhang[1,3,4], Yanli Liu[1,3,4], Zhendong Tian[1,2,3,4§]

1. National Key Laboratory for Germplasm Innovation & Utilization of Horticultural Crops, Huazhong Agricultural University (HZAU), Wuhan 430070, China

2. Hubei Hongshan Laboratory (HZAU), Wuhan 430070, China

3. Key Laboratory of Potato Biology and Biotechnology (HZAU), Ministry of Agriculture and Rural Affairs, Wuhan 430070, China

4. Potato Engineering and Technology Research Center of Hubei Province (HZAU), Wuhan 430070, China

§ Authors for correspondence: tianzhd@mail.hzau.edu.cn

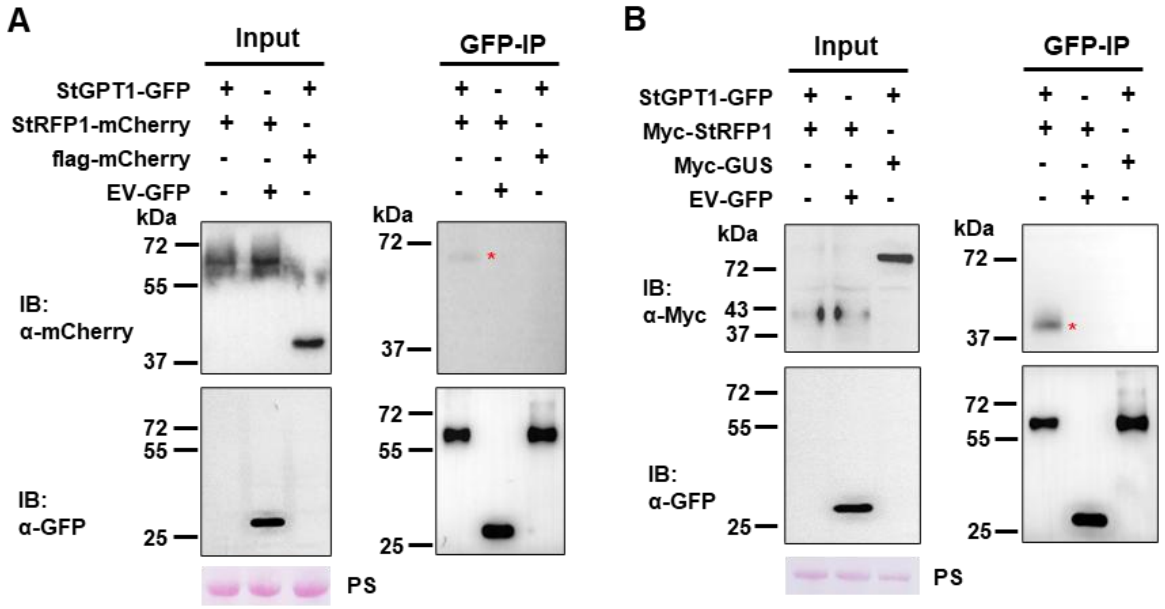

Figure S1. Independent immunoblot replicates demonstrate that StRFP1 interacts with StGPT1. **A** EV-GFP and flag-mCherry were used as negative controls. **B** EV-GFP and Myc-GUS were used as negative controls. IB, immunoblotting. + indicates expression of constructs in *Nicotiana benthamiana* leaves. Protein size given in kilodalton (kDa), protein loading indicated by Ponceau stain. * indicates target protein band.

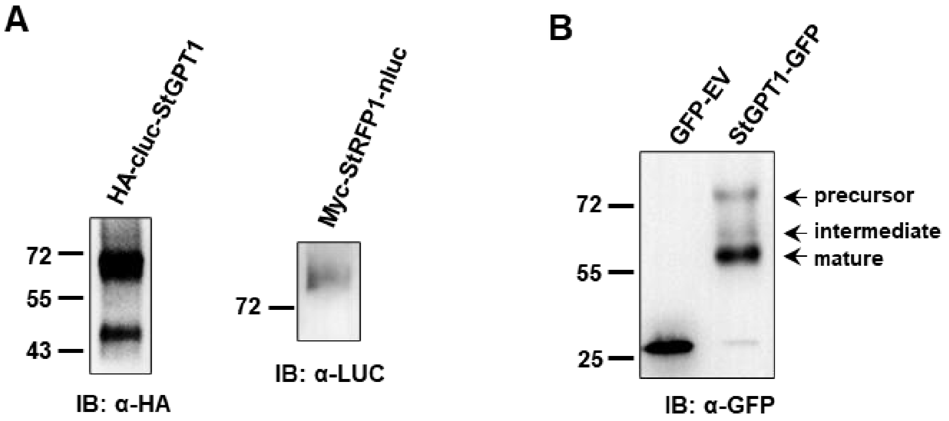

Figure S2. Immunoblots confirm the protein expression of constructs used in LCA and Co-IP. **A** Immunoblots confirm the proteins expression of constructs used in luciferase complementation assay (LCA). **B** Immunoblot shows variants formation of StGPT1-GFP transiently expressed in *N. benthamiana*.

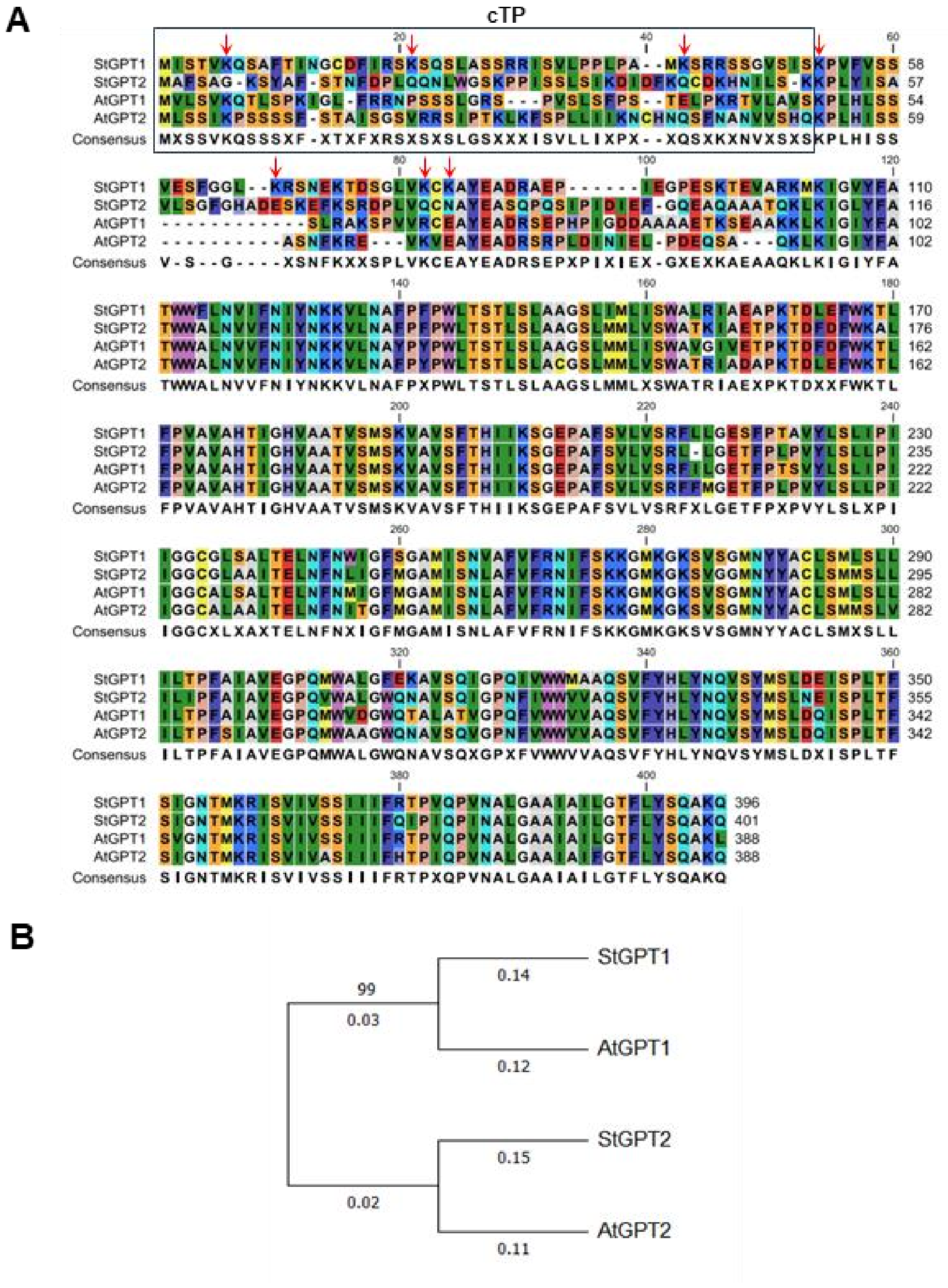

Figure S3. Alignment and phylogenetic tree of potato and *Arabidopsis* GPT1/2. **A** Amino acid alignment of GPTs of *Solanum tuberosum* and *Arabidopsis thaliana*. Alignment was generated in CLC Sequence Viewer 6. Conserved amino acids were indicated with the same color. Lysine residues (K) in the N-terminal StGPT1 region indicated by arrows. **B** The phylogenetic tree was constructed using the Neighbor-Joining method using MEGA11. Bootstrapping was performed with 1000 replicates and values are displayed on branches. StGPT1 (M0ZKH6), StGPT2 (O64911), AtGPT1 (Q9M5A9), AtGPT2 (Q94B38).

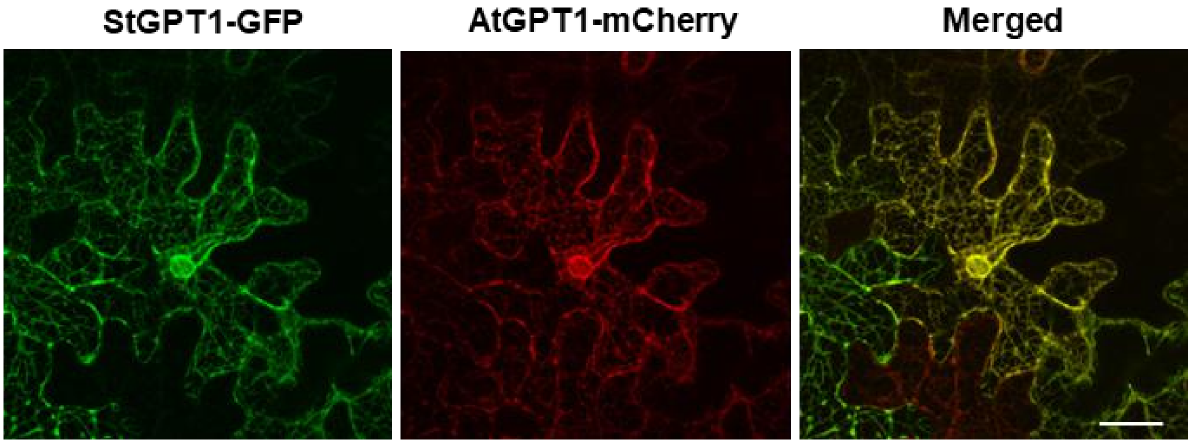

Figure S4. StGPT1 co-localizes with AtGPT1. Co-expression of StGPT1-GFP (left) with AtGPT1-mCherry (center) and a merged image (right). Scale bar is 20 μm.

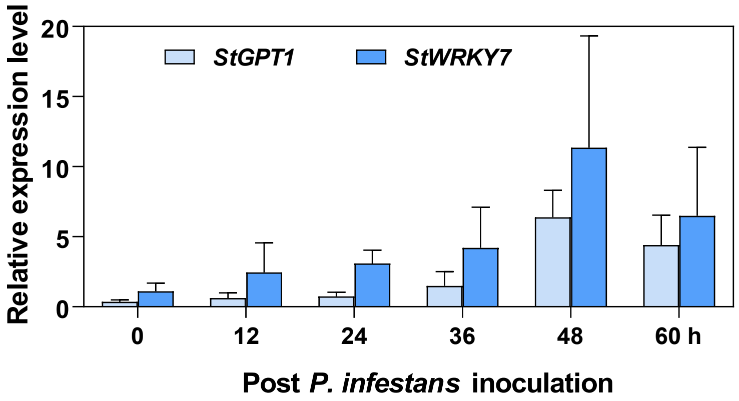

Figure S5. The expression pattern of *StGPT1* responds to *P. infestans* infection. The relative expression levels of *StSGPT1* and *StWRKY7* in response to inoculation with *P. infestans* isolate 88069 in potato 'Désirée'. Leaves were collected at 0, 12, 24, 36, 48 and 60 h post inoculation. Total RNAs were extracted and subsequently reverse transcribed into cDNA. The transcription level was detected by qRT-PCR. The data represent three biological repeats. Error bars indicate ± SE.

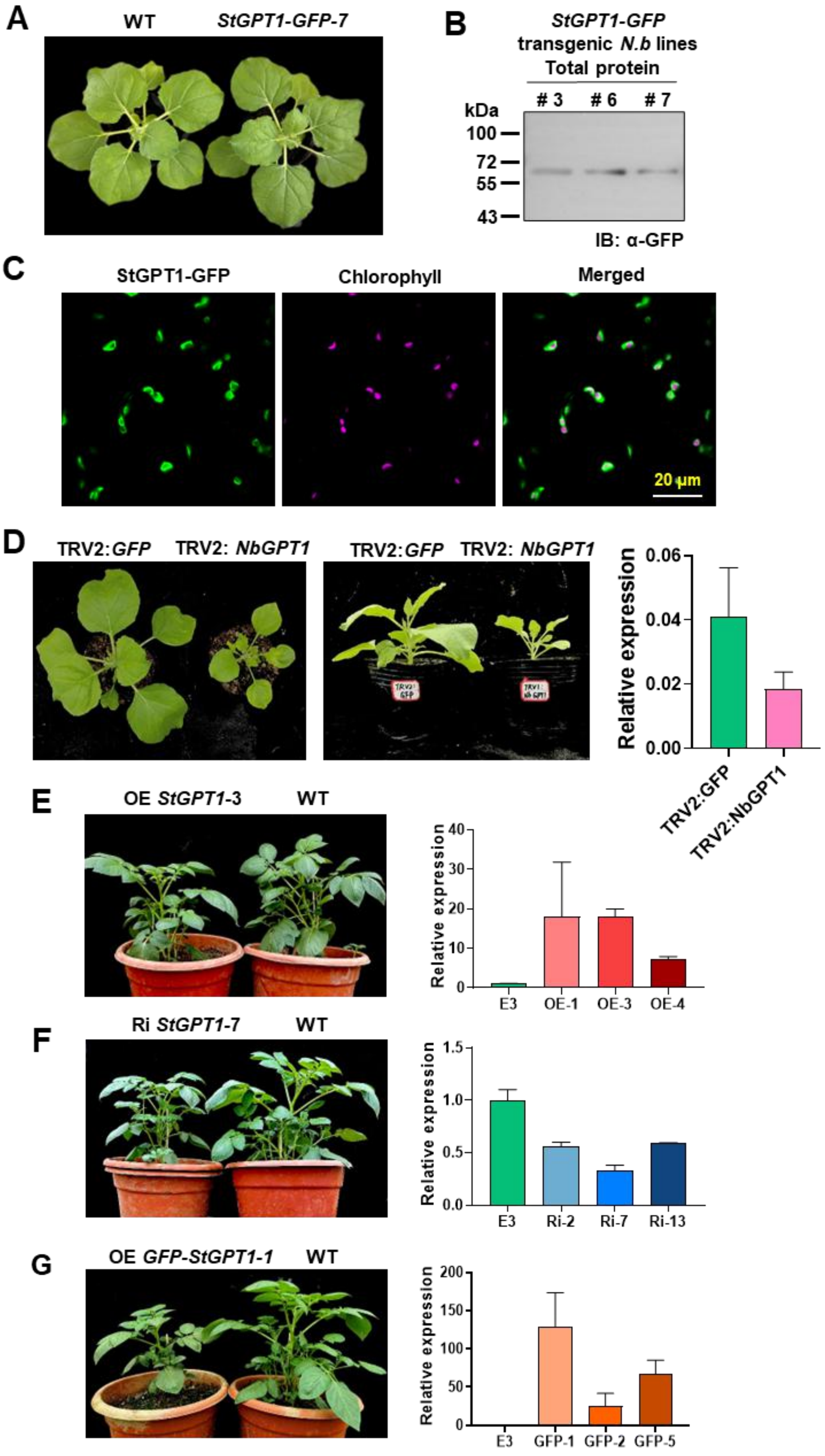

Figure S6. Information of *StGPT1* transgenic lines and TRV2: *NbGPT1* lines. **A** Representative image showing there was no significant phenotype difference between *StGPT1* transgenic *N. benthamiana* and wild-type. **B** Immunoblot shows

that the protein expression in three *StGPT1-GFP* transgenic *N. benthamiana* lines (# 3, # 6, # 7). Total proteins were extracted (lane 1-3) and western blot was performed by specific GFP antibody. **C** StGPT1-GFP fusion protein localizes to chloroplast in transgenic *N. benthamiana* leaves. Bar is 20 μm. **D** Silencing *NbGPT1* in *N. benthamiana* leads to stunted growth. The right bar graph shows the relative expression level of *NbGPT1* in VIGS plants. **E-G** The phenotypes (left) and the relative expression levels of *StGPT1* (right) in overexpression (OE) potato lines (E), RNAi-silenced lines (F) and *GFP-StGPT1* lines. Representative RNAi-*StGPT1*-7 and *GFP-StGPT1-1* transgenic potato lines show stunted growth phenotype. The wild-type ‘E3’ was used as a control. The data represent three biological repeats. Error bars indicate ± SE.

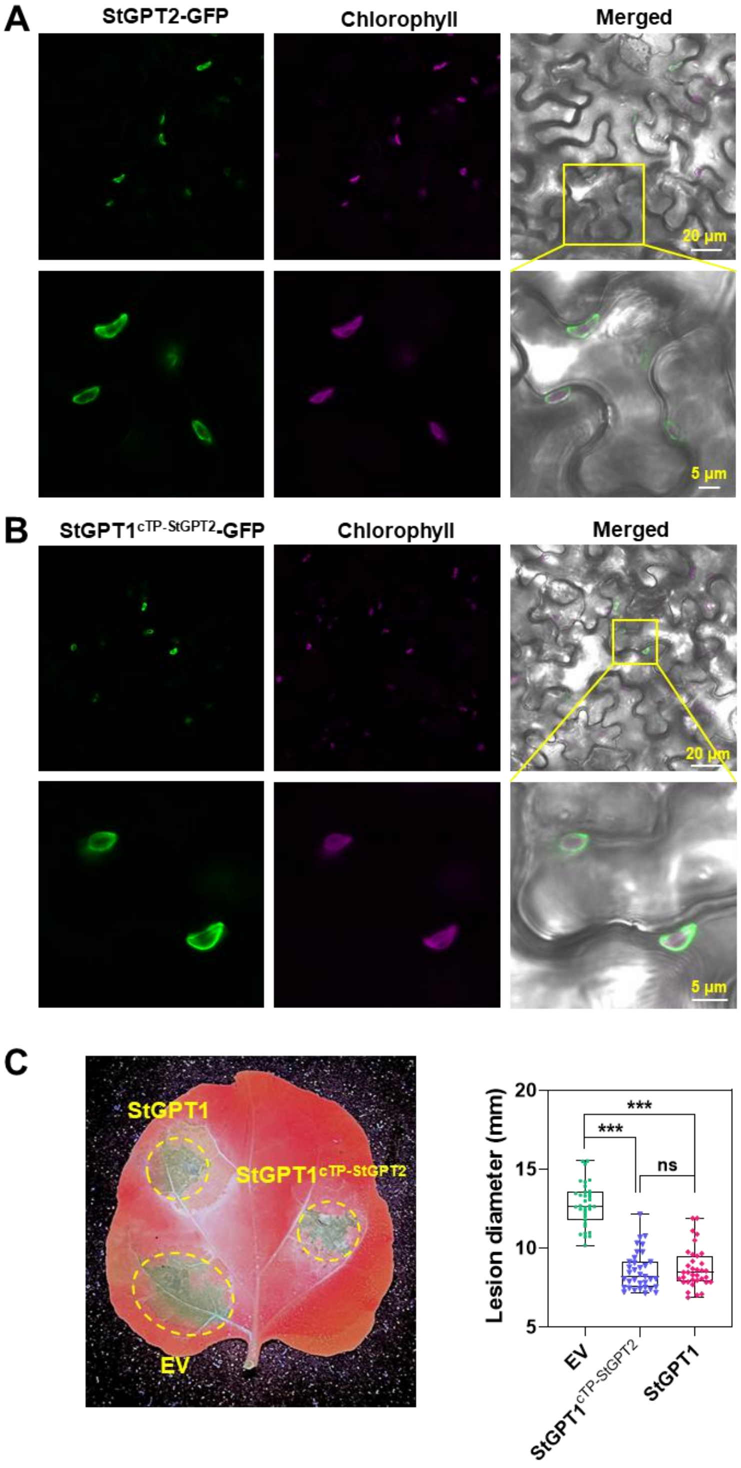

Figure S7. StGPT1$^{cTP\text{-}StGPT2}$ also has the function for resistance against *P. infestans*. **A-B** Confocal images show StGPT2 and StGPT1$^{cTP\text{-}StGPT2}$ localized to the chloroplasts. GFP fluorescence indicates StGPT2/StGPT1$^{cTP\text{-}StGPT2}$, pink indicates spontaneous chloroplast fluorescence. **C** Representative image showing diameters of disease lesions on *N. benthamiana* leaves at 5 dpi with *P. infestans* isolate 88069. Plot graph showing differences in lesion diameters. One-way ANOVA was used for

statistical analysis, *** $p < 0.001$, three independent repeats, n = 35. Error bars indicate ± SE.

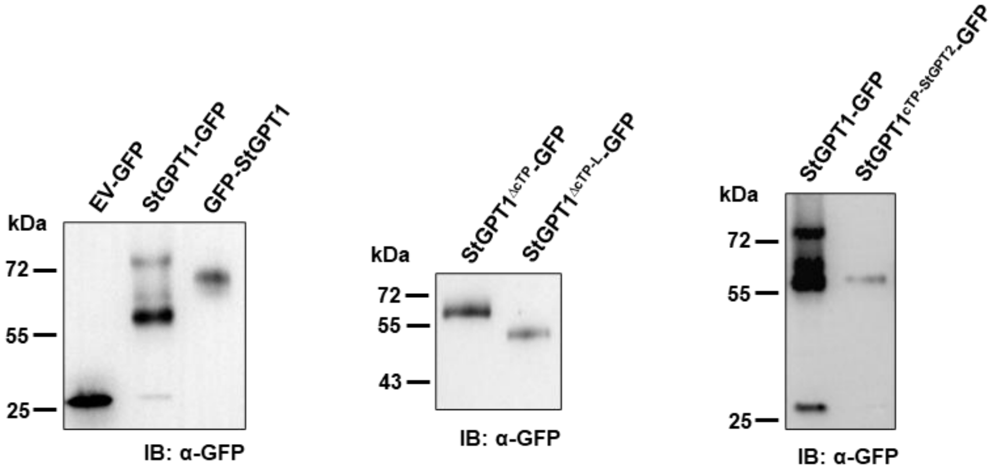

Figure S8. Immunoblots confirm protein expression of the constructs agro-infiltrated in *N. benthamiana*. Immunoblot shows the proteins expression of EV-GFP, StGPT1-GFP and GFP-StGPT1 and StGPT1$^{\Delta cTP\text{-}GFP}$, StGPT1$^{\Delta cTP\text{-}L}$-GFP, StGPT1$^{cTP\text{-}StGPT2}$.

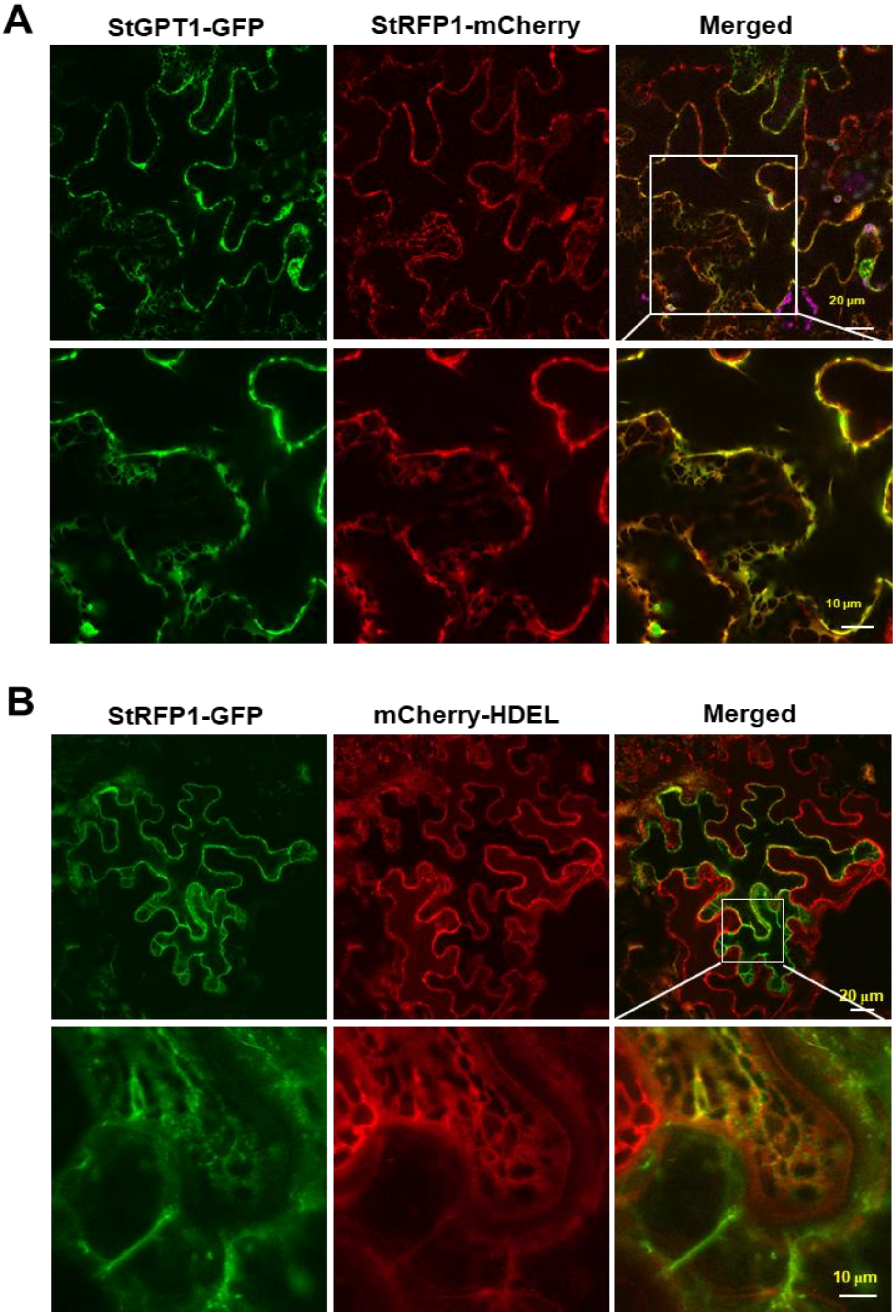
A
StGPT1-GFP
StRFP1-mCherry
Merged
20 µm
10 µm
B
StRFP1-GFP
mCherry-HDEL
Merged
20 µm
10 µm

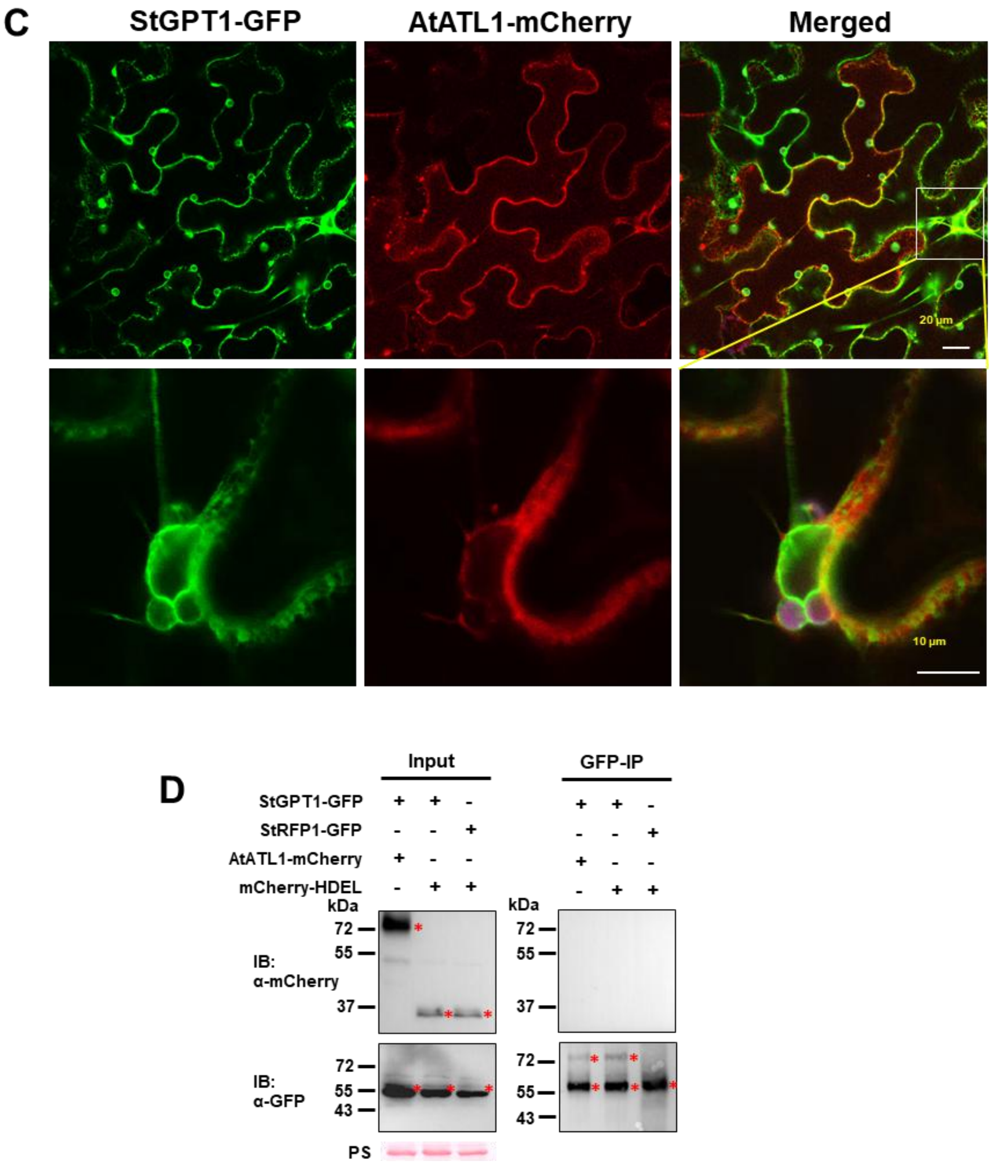

Figure S9. Supplementary confocal images show StGPT1 co-localized with StRFP1 on the ER. **A** Amplified image shows StGPT1-GFP and StRFP1-mCherry co-localized on the ER. **B** StRFP1-GFP did not co-localize with the ER marker mCherry-HDEL. **C** StGPT1-GFP and AtATL1-mCherry did not co-localize. Constructs were co-expressed in *N. benthamiana*. **D** Co-IP assay demonstrated that StGPT1 did not interact with AtATL1 or mCherry-HDEL, StRFP1 did not interact with mCherry-HDEL. * indicates the target protein band.

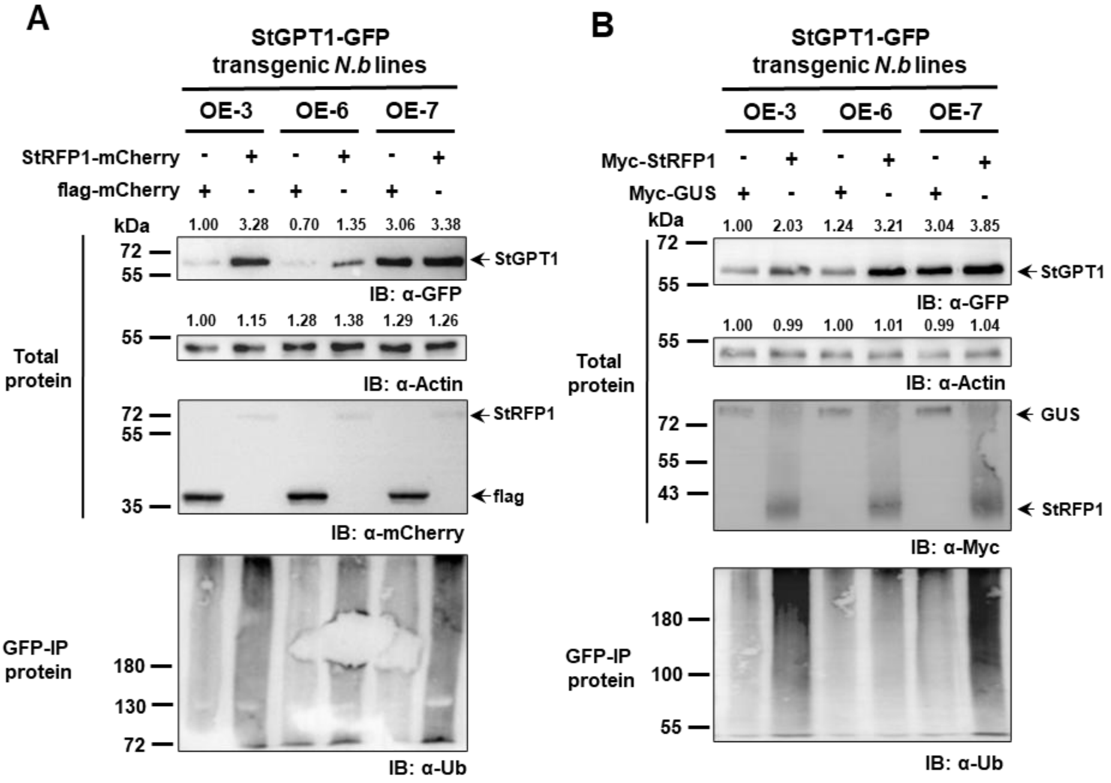

Figure S10. Independent immunoblot replicates show that mature-StGPT1 accumulated significantly in the presence of StRFP1 in transgenic *N. benthamiana* leaves. Expression of constructs is indicated by +. Protein size is given in kilodalton (kDa). Flag-mCherry (A) and Myc-GUS (B) were used as negative controls.

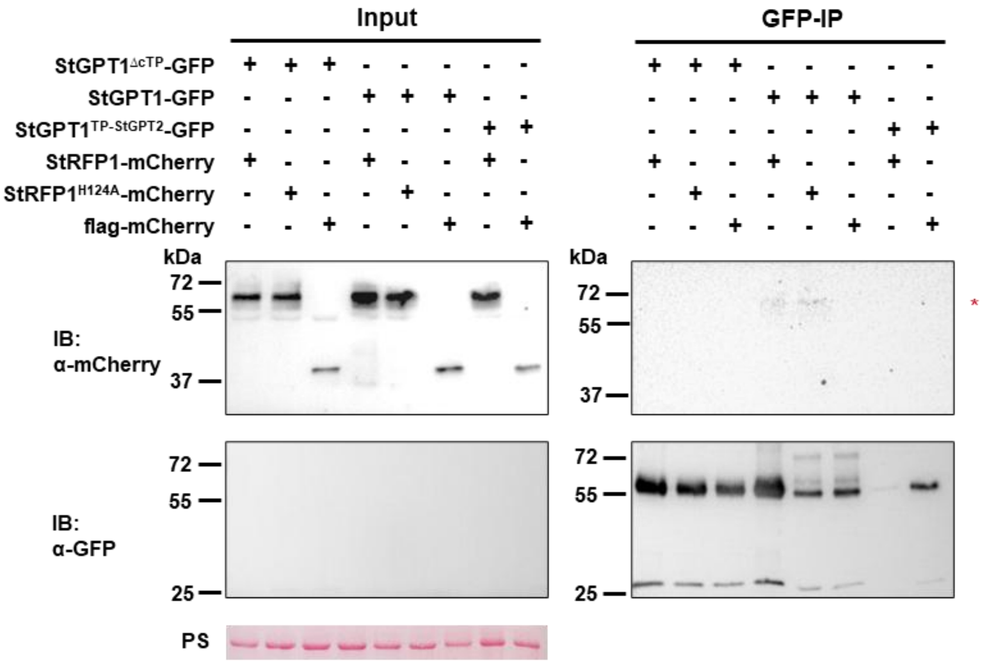

Figure S11. StGPT1 interacts with StRFP1 and StRFP1^{H124A}. Total proteins were extracted, followed by IP with GFP-trap beads. Flag-mCherry was used as negative control. IB, immunoblotting. + indicates expression of constructs in *N. benthamiana* leaves. Protein size given in kilodalton (kDa), protein loading indicated by Ponceau stain. * indicates target protein band.

Table S1. List of primers used in this work.

Primer		Sequence (5'-3')
pBT3-STE-StGPT1-F	F	ATTAACAAGGCCATTACGGCCATGATCTCTACAGTGAAGCAATCG
	R	AACTGATTGGCCGAGGCGGCCTCATTGTTTTGCCTGTGAGTACAAG
MYC-StRFP1-nluc	F	GAAGATTACGCCGGATCCATGGGAAGTGGTAAATTAGGTG
	R	GTACGAGATCTGGTCGACCTAGCTCTGGCCCCTGCTTCCT
HA-cluc-StGPT1	F	GCTGATTACGCCGGATCCATGATCTCTACAGTGAAGCAATCG
	R	AAAGCTCTGCAGGTCGACTCATTGTTTTGCCTGTGAGTACAAG
StGPT1-GFP	F	TGAGCCACCATGGCTGGATCCATGATCTCTACAGTGAAGCAATCG
	R	CTTGCTCACCATCCGCTCGAGTTGTTTTGCCTGTGAGTACAAG
AtGPT1-mCherry	F	CATTTGGAGAGGACACGCTCGAGATGGTTTTATCGGTGAAGCAAAC
	R	TCGCCCTTGCTCACCATGAATTCGAGCTTTGCCTGGGAATAC
StRFP1-GFP	F	TGAGCCACCATGGCTGGATCCATGGGAAGTGGTAAATTAGGTG
	R	CTTGCTCACCATCCGCTCGAGGCTCTGGCCCCTGCTTCCT
StRFP1-mCherry	F	CATTTGGAGAGGACACGCTCGAGATGGGAAGTGGTAAATTAGGTG
	R	TCGCCCTTGCTCACCATGAATTCGCTCTGGCCCCTGCTTCCT
TRV2-NbGPT1	F	AAGGTTACCGAATTCTTCAGCAGTTAGTGGATTTAGACAT
	R	CTCGGTACCGGATCCAGCTGCAGCAACTTGAG
pHellsgate8-StGPT1-1	F	TTTGGAGAGGACACGCTCGAGGTTTTGGTTTCAAGGTTCCTC
	R	TGGGGTACCGAATTCCTCGAGGCAAGCATAGTAGTTCATCCC
pHellsgate8-StGPT1-2	F	GATAAGCTTGGATCCTCTAGAGTTTTGGTTTCAAGGTTCCTC
	R	TCATTAAAGCAGGACTCTAGAGCAAGCATAGTAGTTCATCCC
GFP-StGPT1	F	ATTACGCCGAGGTCATGATCTCTACAGTGAAGCAATCG
	R	TAGGGAAGAGGTCATTGTTTTGCCTGTGAGTACAAG
StGPT1$^{\Delta cTP}$-GFP	F	TGAGCCACCATGGCTGGATCCATGAAGCCTGTTTTTGTTTCTTCTG
	R	CTTGCTCACCATCCGCTCGAGTTGTTTTGCCTGTGAGTACAAG
StGPT1$^{\Delta cTP\text{-}L}$-GFP	F	TGAGCCACCATGGCTGGATCCATGATCGGTGTTTATTTTGCTACTTGG
	R	CTTGCTCACCATCCGCTCGAGTTGTTTTGCCTGTGAGTACAAG
qPCR-StGPT1	F	TTGGCTTCTTCACGCCGTAT
	R	TCCTTCAATCGGTTCAGCCC
qPCR-StWRKY7	F	CCAACTGGAAGCAACAACAA
	R	CCTGATTAGAATGATTAGCCAACA
qPCR-NbGPT1	F	CAAAATTGCCCTGCAAAAGTGTAAT
	R	GCCCAATCTTGAGCTTCTGAGC
Actin	F	CAGAAAGGACCTCTACGGTAACAT
	R	TCTGTGGACGATGGACGGAC

Ri-StGPT1 specific sequence

GTTTTGGTTTCAAGGTTCCTCTTGGGAGAGTCATTCCCTACCGCCGTTTACTTATCTCTTATCCCCATCATCGGTGGT
TGTGGTCTTTCTGCTCTTACAGAGTTGAACTTCAACTGGATTGGTTTCTCGGGGGCTATGATCTCGAATGTGGCATTT
GTCTTCAGAAATATATTCTCCAAGAAGGGTATGAAGGGGAAGTCTGTTAGTGGGATGAACTACTATGCTTGC

TRV2-NbGPT1 specific sequence

TTCAGCAGTTAGTGGATTTAGACATGTTGATGAATCAAAAGAGTCAACATCTAGGGACAAATTAGTCCAGTGCAATGC
CTATGAAGCAAGCAGACCACAGTCAATACCAATTAGCATTGATTTTGATAAAGAAACTCAAGTTGCTGCAGCT

Table S2. Putative interaction proteins identified from the Y2H library.

No.	Appearance	Gene Name	NCBI ID
1-1	1	*Solanum tuberosum* syntaxin-43-like (LOC102593977)	XM_006344657.1
1-5	1	*S. tuberosum* phosphatidylinositol 4-kinase gamma 4-like (LOC102583460)	XM_006339653.2
1-6	1	*S. tuberosum* glucose-6-phosphate/phosphate translocator 1 (LOC102578523)	XM_006365979.2
1-7	1	*S. tuberosum* uncharacterized LOC102603652	XM_006354104.2
1-9	2	*S. tuberosum* proton pump-interactor 1 (LOC102591229)	NW_006239025.1
1-11	2	*S. tuberosum* sugar carrier protein C (LOC102596883)	XM_006347081.2
2-3	1	*S. tuberosum* cytochrome b561 and DOMON domain-containing protein At5g47530-like (LOC102589404)	XM_006342206.2
2-4	1	*S. tuberosum* serine protease inhibitor 5-like (LOC102599070)	XM_006351452.2
2-5	1	*S. tuberosum* uncharacterized LOC102589648	NW_006239526.1
3-1	1	*S. tuberosum* ras-related protein RABE1c (LOC102582485)	NW_006239356.1
3-3	1	*S. tuberosum* tetraspanin-8-like (LOC102589183)	XM_006343502.2
3-5	1	*S. tuberosum* RRP12-like protein (LOC102590327)	XM_006353716.2
4-9	1	*S. tuberosum* calreticulin (LOC102603479)	XM_006344690.2
4-10	1	*S. tuberosum* serine-threonine kinase receptor-associated protein-like (LOC102586216)	XM_006341607.2
4-12	3	*S. tuberosum* uncharacterized LOC102580932	NW_006239346.1
5-9	1	*S. tuberosum* protein CURVATURE THYLAKOID 1B, (LOC102602283)	XM_006352563.2
5-12	1	*S. tuberosum* uncharacterized LOC102605286	XM_006346216.2
6-1	1	*S. tuberosum* probable aquaporin PIP2-2 (LOC102589206)	XM_006345744.2
7-7	2	*S. tuberosum* bidirectional sugar transporter N3	KU686986
8-9	1	*S. tuberosum* cytochrome b5 isoform E (LOC107061245)	XM_015310118.1
9-2	1	*S. tuberosum* amino acid transporter ANTL1-like (LOC102601768)	XM_006340802.2
9-3	1	*S. tuberosum* squalene synthase-like (LOC102582345)	XM_015314921.1
10-4	1	*S. tuberosum* serine SHMT hydroxymethyl transferase (LOC102591486)	XM_015313581.1
10-6	1	*S. tuberosum* cultivar Agria aspartic protease inhibitor 5 (API5)	MH686153.1
11-4	1	*S. tuberosum* PGR5-like protein 1B(LOC102584115)	XM_006358321.2
11-5	1	*S. tuberosum* uncharacterized LOC102606139	XM_006339299.2
11-7	1	*S. tuberosum* protein Asterix (LOC102578671)	XM_006354696.2
11-8	1	*S. tuberosum* lysine histidine transporter 1-like (LOC102596930)	XM_006338609.2
11-10	1	*S. tuberosum* cationic peroxidase 1-like (LOC102601606)	XM_006349506.2

1	1	*S. tuberosum* long chain acyl-CoA synthetase 8 (LOC102603429)	NW_006239173.1
2	1	*S. tuberosum* scopoletin glucosyltransferase (LOC102583517)	XM_006346326.2
3	1	*S. tuberosum* uncharacterized LOC102601839	NW_006239057.1
4	1	*S. tuberosum* ras-related protein RABA2a (LOC102585266)	XM_006349248.2
5	1	*S. tuberosum* uncharacterized LOC102598318	XM_006358435.2
6	1	*S. tuberosum* NAC domain-containing protein 91 (LOC102605066)	XM_006347254.2
7	1	*S. tuberosum* chorismate synthase 2, chloroplastic (LOC102580455)	NW_006239217.1
